# Long-Term Effects of a Randomized Maternal Education Trial in Rural Uganda: Implications for Child Oral Health

**DOI:** 10.4269/ajtmh.22-0248

**Published:** 2022-09-06

**Authors:** Marit S. Engh, Grace K. M. Muhoozi, Moses Ngari, Anne B. Skaare, Ane C. Westerberg, Per Ole Iversen, Ingvild J. Brusevold, Prudence Atukunda

**Affiliations:** ^1^ Institute of Clinical Dentistry, Faculty of Dentistry, University of Oslo, Oslo, Norway;; ^2^ Department of Human Nutrition and Home Economics, Kyambogo University, Kampala, Uganda;; ^3^ The Childhood Acute Illness & Nutrition Network (CHAIN), Nairobi, Kenya; KEMRI/Wellcome Trust Research Programme, Kilifi, Kenya;; ^4^ School of Health Sciences, Kristiania University College, Oslo, Norway;; ^5^ Division of Obstetrics and Gynecology, Oslo University Hospital, Oslo, Norway;; ^6^ Department of Nutrition, Institute of Basic Medical Sciences, University of Oslo, Oslo, Norway;; ^7^ Department of Haematology, Oslo University Hospital, Oslo, Norway;; ^8^ Division of Human Nutrition, Stellenbosch University, Tygerberg, South Africa

## Abstract

The aim was to examine oral health among 5–6-year-old children whose mothers participated in a 6 months’ cluster-randomized education trial in rural Uganda starting when their children were 6–8 months old. The education focused on nutrition, oral hygiene, and child stimulation. In the current follow-up study, 357/511 (70%) children from the original trial were available for data collection (200 in the intervention and 157 in the control group). Molar caries was assessed on intraoral photographs. Children and/or caregivers answered a WHO health questionnaire for collection of oral data. Dental practices were compared between the intervention and control group using multilevel mixed effect logistic regression accounting for clustering. The children in the intervention group had less caries compared with the control group: 41% versus 60% (odds ratio [OR] 0.46; 95% confidence intervals [CI] 0.24–0.86, *P* = 0.02). The use of toothbrush to clean teeth was more frequent in the intervention than in the control group: 66% versus 38% (OR 3.39; 95% CI 1.54–7.45, *P* = 0.003), as was high teeth-cleaning frequency: 74% versus 62% (OR 1.72; 95% CI 1.09–2.69, *P* = 0.02). Self-reported problems such as toothache (10% versus 19%), difficulty biting (12% versus 24%) and chewing food (8.5% versus 18%) were significantly less frequent among children in the intervention compared with the control group. No significant differences were found in dietary habits. Our data shows that an educational intervention adjusted to a low-resource setting, provided in infancy, resulted in improved oral hygiene and reduced development of dental caries among children aged 5–6 years.

## INTRODUCTION

Despite improvements in the oral health of populations globally, oral diseases remain a major health problem with negative impact on the affected individual, their families, and the society.^1^^–^^3^ By definition of the World Dental Federation and later adopted by the World Health Organization (WHO): “Oral health is multi-faceted and includes the ability to speak, smile, smell, taste, touch, chew, swallow and convey a range of emotions through facial expressions with confidence and without pain, discomfort and disease of the craniofacial complex (head, face, and oral cavity).”^4^

According to WHO, 3.5 billion people worldwide suffer from oral diseases, untreated dental caries being the most common.^5^ In 2017, more than 530 million children had untreated caries in deciduous teeth,^5^ indicating a lack of oral disease prevention and insufficient care for children worldwide, particularly among the poor and vulnerable groups.^6^^,^^7^ In high-income countries (HIC), caries prevalence was very high until the 1970s when fluoride products were introduced and caries prevalence declined.^8^^,^^9^ However, in most African countries dental caries was less common in the 1970s than today.^10^^,^^11^ In many low- and middle-income countries (LMICs), sugar consumption has increased while dental hygiene and fluoride products are not commonly available; therefore, the risk and burden of caries has increased the in last 50 years.^10^ In Africa, dental treatment is not accessible or affordable for a large proportion of the population.^10^ In line with this, the global burden of disease for dental caries remains high and unchanged in the last three decades,^2^ with a high proportion of unmet dental treatment needs.^5^^,^^6^

Improvement on oral health will contribute toward SDG3 (UN’s Sustainable development goals: promoting good health and well-being) and poor oral health has been acknowledged as a major health problem by WHO.^12^ In 2021, The World Health Assembly approved a historic resolution on oral health, acknowledging the common risk factors, most importantly diet and hygiene, to other noncommunicable diseases such as cardiovascular diseases, diabetes, cancers, pneumonia, obesity, and premature birth.^13^ It was an important milestone in getting oral health integrated in overall health. The resolution calls for effective programs of preventive approaches from all member states to tackle oral diseases and other noncommunicable diseases with similar risk factors.^14^ Many countries do not have accessible preventive programs for children.

Dental caries is the destruction of dental hard tissues by acidic products formed by bacteria in dental biofilm.^15^^,^^16^ The disease has a multifactorial etiology and is largely preventable. It is widely accepted that a diet high in sugar^17^ and poor oral hygiene are factors that contribute to the development of the disease. On the other hand, topical use of fluoride increases the tooth’s resistance against bacterial acids by lowering the critical pH at which the dental enamel hydroxyapatite starts to dissolve. Therefore, regular mechanical removal of the dental biofilm with, for example, a toothbrush and fluoridated toothpaste and reduction in sugar intake is essential in the prevention of the disease.^16^

Traditional oral health practices are common in many LMICs, including Uganda. For example, the removal of primary canine tooth buds in infants may cause severe illness or even death,^10^^,^^18^^,^^19^ and is a form of infant oral mutilation.^20^ The long-term impacts on the dentition are damage to the underlying permanent tooth bud or adjacent permanent tooth, resulting in dental abnormalities, tooth loss, and dental eruption complications.^18^^,^^21^ The tooth bud believed to cause problems is known by different names depending on the region and language spoken including *false teeth*, *killer teeth*, *nylon teeth,* and *worm/maggot teeth*.^21^ *Ebiino* is the term used in several districts of Southwestern Uganda.^20^

Recent data from cross-sectional studies have revealed high prevalence of caries among Ugandan children and a need for better treatment options to avoid future dental complications.^22^^,^^23^ Since oral diseases are mostly behavior- and lifestyle-related, simple and inexpensive interventions could be an efficient way to improve children’s oral health in low-resource settings such as rural Uganda.^24^ The “Child Nutrition and Development Study” (CHNUDEV) was a randomized controlled trial (RCT) conducted in Southwestern Uganda in 2013–2014.^25^ The main aim of that RCT was to study the effect on growth among children aged 20–24 months of a maternal education intervention focusing on nutrition, oral health, hygiene, and child stimulation.^26^ In a follow-up study of the children at age 36 months, we found that the intervention markedly 1) improved teeth-cleaning, 2) reduced the occurrence of caries, and 3) reduced removal of false teeth (*ebiino*).^27^ The aim of the current follow-up study was to determine whether these positive outcomes on oral health were sustained in the primary molars that erupted after the intervention, assessed when the children started at school, that is, when they were 5–6 years old. This provided a unique opportunity to perform a long-term follow-up of a maternal education trial, which is rare in LMICs in general, in particular in Sub-Saharan Africa.

## MATERIALS AND METHODS

### Overview of the original RCT.

Details of the original cluster-RCT are available in the Supplemental material (Supplemental Appendix S1) and elsewhere.^26^^,^^27^ The primary outcome was linear growth while development outcomes and oral (dental) health were secondary outcomes. Briefly, proportionate sampling was used to select subcounties (clusters) in two districts of Southwestern Uganda (six in Kabale district and four in Kisoro district). All villages in each subcounty (intervention or control) were listed alphabetically and computer-generated random numbers by the study statistician were then used to obtain the villages, and finally complete enumeration was used to obtain participating households. Intervention villages did not share common geographical boundaries with control villages to prevent “contamination” of intervention-contents between the two study groups. Exclusion criteria were congenital malformations or physical handicap among children that would influence food intake, growth, and mental or brain illness as evidenced by mother or health worker.

### Contents of the maternal education intervention.

The maternal education intervention emphasized nutrition, oral health, hygiene, and child stimulation as described in the Supplemental material (Supplemental Appendix S1) and as previously detailed.^26^ The intervention was implemented when children were 6–8 months old and lasted 6 months. Each group (*N* = 8–12) of mothers received three main education sessions (with a nutrition education team) followed by monthly village meetings. Thereafter, booster sessions were provided every third month until the age of 36 months (Supplemental Appendix S1). The oral hygiene education took place when the children were 12–16 months. The intervention group received routine healthcare and the education intervention while the control group received only routine healthcare. Our strategy with the intervention was to promote behavior change through providing information and prompt practice (demonstrations).

### Collection of oral data.

Oral data was initially collected when the children were 20–24 months, and at 36 months.^26^^,^^27^ In the current follow-study among the children aged 5–6 years, we used an oral health questionnaire for collection of data, and close-up intraoral photographs were taken of the children’s posterior teeth to appraise the occurrence of dental caries.

#### Oral health questionnaire.

The WHO—oral health questionnaire, children’s version was used to collect data.^28^ Unfamiliar food on the questionnaire were replaced with the common cariogenic foods in the area, for example, Brushera with sugar and sugar canes. Two research assistants who spoke the local language fluently, performed the face-to-face interviews with each child and their primary caregiver and filled in the questionnaire. Details of the questions can be found in the Supplemental material (Supplemental Appendix S2).

#### Intraoral photographs.

The research assistants, trained to take photos, took four close-up intraoral photographs of the maxillary and mandibular lateral segments of each child, using a digital camera (Canon EOS 60D, Canon INC., Japan) fitted with a macrolens (Canon Macro Lens EF 100 mm 1:2,8 USM, Japan) and a ring flash (Canon Ring Lite MR-14EX, Japan). Intraoral mirrors were used in the upper jaw. The photographs were later evaluated by three experienced pediatric dentists (MSE, IJB, and ABS) to determine the occurrence of dental caries on first and second primary molar, and first permanent molar if erupted. Dental caries was recorded using the WHO definition of caries,^29^ and unmistakable cavities progressing into the dentin were registered as caries. The early stages of dentin caries are not possible to identify on intraoral photographs and without a clear cavity the tooth was registered as sound. We distinguished between “less severe cavitation” and “severe decay.” “Less severe cavitation” was used when we found a well-defined cavity affecting one tooth surface. “Severe decay” described teeth with more than one surface with caries including severely damaged teeth, teeth with only root left and missing primary teeth assumed extracted due to caries. In the analysis, we collapsed this variable into “sound/less severe decay” versus “severe decay.” Since the first permanent molars usually erupt at age 6–7 years, a missing first permanent molar was registered as unerupted because of young age.

Signs of possible removed *ebiino* were recorded if the photographs, by chance, showed canine area. The clinical signs after the procedure were missing primary canine, hypoplastic primary canines, and enamel opacity (localized developmental defect) involving the buccal surface or incisal edge of a primary canine, in addition to absence or dental anomalies in adjacent teeth.

The close-up intraoral photographs were of uneven quality and pictures of some of the children were missing. When the photo had poor quality, food leftovers or plaque covered the tooth surfaces, the tooth was registered as “unreadable.”

All the evaluations/scorings were done blinded to study group allocation. The examiners’ evaluations were calibrated by discussion and assessments. The photographs of 52 children were evaluated twice, and intraexaminer agreement was excellent (Cohen’s unweighted kappa value was 0.88).^30^

### Ethical approvals.

The study was reviewed by the Makerere University School of Public Health, Higher Degrees Research and Ethics Committee, and was approved by the Uganda National Council for Science and Technology (No. HS 1809), after being reviewed by The AIDS Support Organisation Research Ethics Committee (No. TASOREC/06/15-UG-REC-009). The Norwegian Regional Committee for Medical and Health Research Ethics also approved the study (No. 2013/1833). The consent form was translated into the local language, and all participants gave written or thumb-printed, informed consent. The original RCT was registered at ClinicalTrials.gov (#NCT02098031).

### Statistical methods.

Data analyses followed intention-to-treat, where all children with data were included and reported in line with the CONSORT guidelines.^31^ In the current follow-up study, with a reported occurrence of dental caries in primary molars at 60% among the control group, a total sample size of 350 children (175 in each arm) was adequate to detect a reduction in dental caries by at least 50% (odds ratio [OR] of 0.5) with a power of 80%, a design factor of 1.5 (calculated using intracluster correlation coefficient of 0.01 in the original RCT) and a two-tailed alpha of 0.05.

All continuous variables were summarized as either means (standard deviation [SD]) or median (interquartile range [IQR]) depending on the underlying distribution. All categorical variables are reported as frequency and their proportions. All comparisons performed were cluster-adjusted to account for the study design of the original RCT. All the 357 children included in the study had complete data on all variables of interest. The few missing responses (< 3%) on self-reported problems related to status of the teeth and oral hygiene, were assumed not to be missing at random and coded as “do not know/unknown”. These are included in the analyses as separate categories. To compare dental practices grouped as binary between the intervention and control group, multilevel mixed effect logistic regression models with unstructured variance-covariance structure and cluster as a random effect component were used. Cluster adjusted odds ratios (aOR) and their 95% confidence intervals (CI) were reported as measure of effect. Statistical significance was set at *P* < 0.05. Statistical analyses were performed using Stata version 17.0 (StataCorp, College Station, TX).

## RESULTS

In the current follow-up study, we managed to include 357 (70%) of the 511 children in the original RCT; 157 (63%) and 200 (76%) randomized to the control and intervention group, respectively. At baseline (start of RCT), their mean (SD) age was 7.4 (1.0) and 7.4 (0.9) months among children randomized to control and intervention group, respectively. The maternal median (IQR)] age in this follow-up cohort was 27 (22–30) and 25 (21–30) years at baseline among the control and intervention group, respectively. At the same time, the household median (IQR) poverty score was 49 (40–56) and 49 (40–57) among control and intervention group, respectively. The distribution of baseline characteristics were well balanced between the two study groups in the current follow-up study and did not differ significantly from those collected for the original RCT (Table 1).^26^ From start of the RCT until the current follow-up study we have not experienced any intervention-related harm in any of the two study groups.

**Table 1:** Study population characteristics at baseline of the original randomized controlled trial

	Original study (*N* = 511)		Current study (*N* = 357)	
	Control (*N* = 248)	Intervention (*N* = 263)	Control (*N* = 157)	Intervention (*N* = 200)
Children characteristics				
Gender-male	123 (50)	139 (53)	76 (48)	106 (53)
Mean (SD) age (months)	7.3 ± 0.9	7.4 ± 0.8	7.4 ± 1.0	7.4 ± 0.9
Underweight	36 (15)	25 (9.5)	17 (11)	16 (8.0)
Wasting	12 (4.8)	12 (4.6)	5 (3.2)	8 (4.0)
Stunting	70 (28)	55 (21)	41 (26)	34 (17)
Exclusive breastfeeding for 6 months				
Yes	178 (72)	184 (70)	111 (71)	136 (68)
No	70 (28)	79 (30)	46 (29)	64 (32)
Breastfeeding frequency				
≥ 8 times/day	171 (69)	170 (65)	110 (70)	130 (65)
< 8 times/day	77 (31)	93 (35)	47 (30)	70 (35)
Illness at recruitment				
Yes	71 (29)	94 (36)	52 (33)	68 (34)
No	177 (71)	169 (64)	105 (67)	132 (66)
Maternal characteristics				
Median (IQR) maternal age (years)*	26 (22 − 30)	25 (21 − 30)	27 (22 − 30)	25 (21 − 30)
Level of education				
None/primary	166 (70)	173 (66)	108 (69)	132 (66)
Lower secondary	62 (25)	64 (24)	39 (25)	51 (26)
Tertiary	20 (8.1)	26 (9.9)	10 (6.6)	17 (7.7)
Number of biological children				
< 5	184 (74)	187 (71)	113 (72)	138 (69)
≥ 5	64 (26)	76 (29)	44 (28)	62 (31)
Household-level characteristics				
Median (IQR) household head age (years)	30 (25 − 38)	30 (25 − 36)	30 (25 − 38)	30 (25 − 35)
Level of education				
None/primary	138 (56)	138 (52)	93 (59)	115 (58)
Lower secondary	80 (32)	78 (30)	47 (30)	56 (28)
Tertiary	30 (12)	47 (18)	17 (11)	29 (14)
Household size				
3–5	139 (56)	150 (57)	85 (54)	116 (58)
6–10	109 (44)	113 (43)	72 (46)	84 (42)
Median (IQR) poverty score	49 (39 − 57)	49 (40 − 57)	49 (40 − 56)	49 (40 − 57)

Values are *N* (%) unless otherwise stated.

* Seven missing records (four in intervention and three in control group). There were no differences (*P* < 0.05) in any of the characteristics between the two study groups, neither for the original randomized controlled trial cohort nor for the follow-up cohort. Underweight was defined as weight-for-age *z*-score < −2 standard deviations (SD) below the median of the WHO child growth standard; wasting as weight-for-height *z*-score < −2 SD; and stunting as height-for-age *z*-score < −2 SD. IQR = interquartile range.

**Figure 1.**
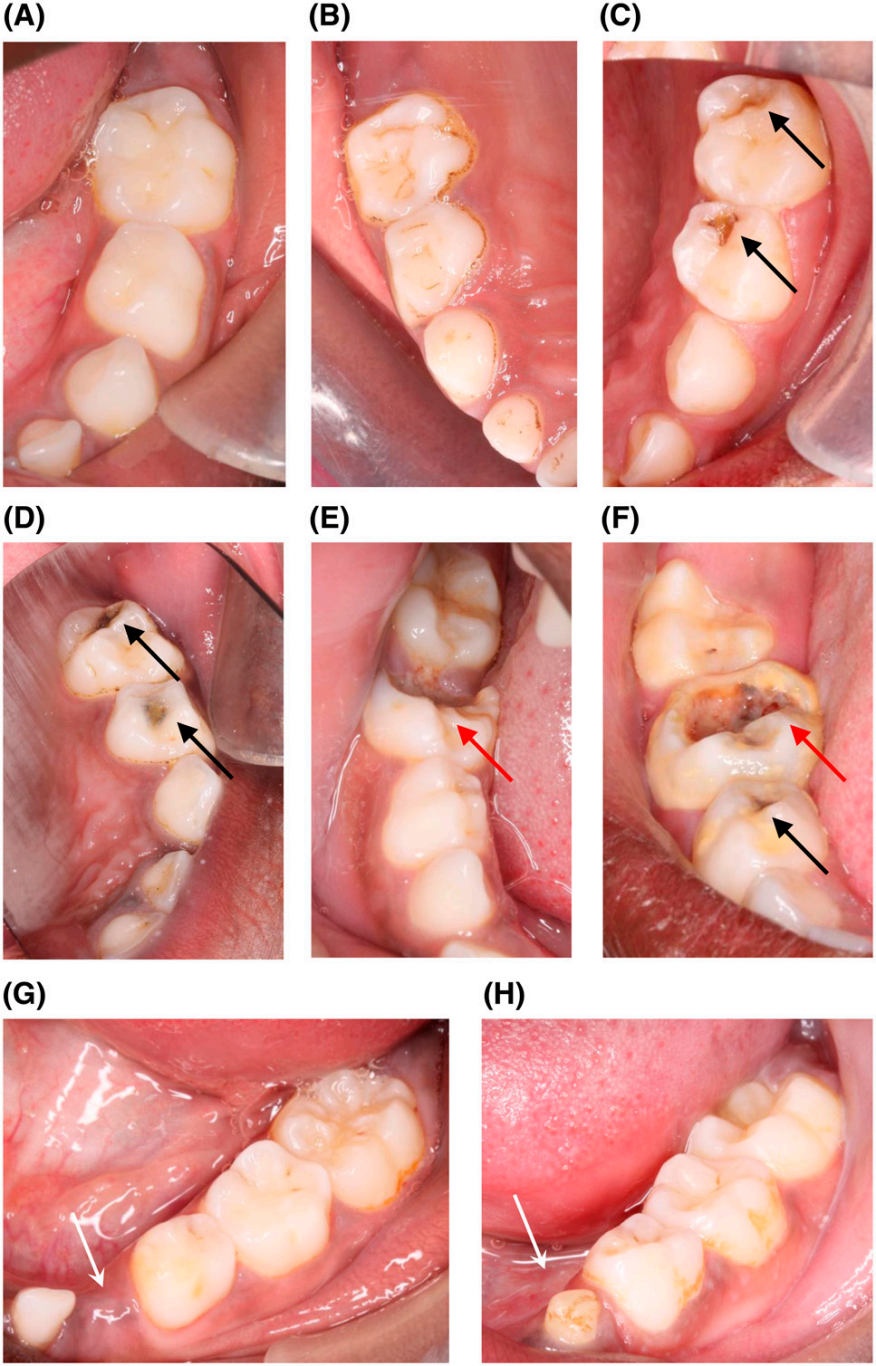
Examples of the different tooth scoring categories. (**A,B**) Sound teeth. Brown stain is discolored biofilm, and not caries. (**C,D**) Less severe caries in primary molars marked with black arrows. (**E,F**) Severe caries in teeth marked with red arrows, and less severe caries in teeth marked with black arrow. g, h: Signs of probable *ebiino* removal marked with white arrows: Missing primary canine (**G**); malformed, hypoplastic primary canine (**H**).

Table 2 shows that the occurrence of caries in primary molars was markedly lower among children in the intervention group, 72 (41%) compared with 90 (60%) among the controls: OR 0.46 (95% CI 0.24–0.86, *P* = 0.02). However, the intervention had only a borderline protective effect against the severity of caries: OR 0.49 (95% CI 0.21–1.11, *P* = 0.09). The data on the number of teeth with decay, possible removed *ebiino*, self-reported dental health, and previous visit to dentist in last year, were not significantly different between the children in the two study groups (Table 2). Examples of photographs showing how caries and possible *ebiino* removal were scored, are shown in Figure 1. Twenty-six children visited a dentist in the previous year. Reported good gum health was significantly higher among children randomized to intervention compared with those in control group (Table 2).

**Table 2:** Oral health status

	Control (*N* = 157)*	Intervention (*N* = 200)*	Odds ratio (95% CI)†	*P* value†
**Dental status based on intraoral photographs**				
Occurrence of caries				
No	61 (40)	104 (59)	Reference	–
Yes	90 (60)	72 (41)	0.46 (0.24–0.86)	0.02
Severity of caries				
Sound/less severe	110 (70)	164 (82)	Reference	–
Severe decay	47 (30)	36 (18)	0.46 (0.21–1.11)	0.09
Number of teeth with caries				
< 4 teeth	121 (80)	159 (90)	Reference	–
≥ 4 teeth	30 (20)	17 (9.7)	0.30 (0.07–1.34)	0.12
Possible removed *ebiino*				
No	119 (79)	158 (90)	Reference	–
Yes	32 (21)	17 (9.7)	0.73 (0.05–9.77)	0.81
**Self-reported oral health status**				
Dental health				
Poor	87 (55)	191 (46)	Reference	
Good	70 (45)	109 (54)	1.49 (0.97–2.27)	0.06
Gum health				
Poor	60 (38)	47 (24)	Reference	
Good	97 (62)	153 (77)	2.06 (1.12–3.78)	0.02
**Dental care**				
Previous visit to dentist in last year				
No	141 (90)	184 (92)	Reference	
Yes	16 (10)	16 (8.1)	0.68 (0.19–2.44)	0.56

* Values are *N* (%).

† The cluster-adjusted odds ratios and *P* values are from multilevel logistic regression models with the cluster as random intercept.

Reported “high frequency” of teeth-cleaning was more common among children in the intervention group (*N* = 147, 74%) compared with controls (*N* = 97, 62%); OR 1.72 (95% CI 1.09–2.69, *P* = 0.02), as was the use of toothbrush (Table 3). However, the frequency of other oral hygiene practices was not significantly different between the two study groups (Table 3). The frequency of tooth ache was less common (*N* = 20, 10%) among children randomized to intervention group compared with control group (*N* = 29, 19%), OR 0.48 (95% CI 0.23–0.98, *P* = 0.04). Other self-reported problems with dental health are shown in Table 4. We found no significant differences in eating and drinking practices between the two study groups (Table 5).

**Table 3:** Oral hygiene practices as reported by the mothers/caregivers

	Control (*N* = 157)*	Intervention (*N* = 200)*	Odds ratio (95% CI)†	*P* value†
**Do you use any of the following to clean teeth or gum?**				
Toothbrush				
No	98 (62)	69 (34)	Reference	–
Yes	59 (38)	131 (66)	3.39 (1.54–7.45)	0.003
Wooden toothpicks				
No	138 (88)	186 (93)	Reference	–
Yes	19 (12)	14 (7.0)	0.41 (0.09–1.87)	0.25
Plastic toothpicks				
No	155 (99)	198 (99)	Reference	–
Yes	2 (1.3)	2 (1.0)	0.77 (0.09–6.67)	0.81
Thread floss				
No	156 (99)	199 (99)	Reference	–
Yes	1 (0.6)	1 (0.5)	0.78 (0.05–12.6)	0.86
Charcoal				
No	143 (91)	184 (92)	Reference	–
Yes	14 (8.9)	16 (8.0)	0.89 (0.42–1.88)	0.76
Chew stick				
No	116 (74)	168 (84)	Reference	–
Yes	41 (26)	32 (16)	0.51 (0.23–1.12)	0.09
Toothpaste				
No	107 (68)	139 (70)	Reference	–
Yes	50 (32)	61 (30)	0.97 (0.48–1.94)	0.92
Toothpaste with fluoride				
No	98 (62)	139 (70)	Reference	–
Yes	35 (22)	46 (23)	0.93 (0.54–1.58)	0.78
Don’t know	24 (15)	15 (7.5)	–	–
**How often do you clean your teeth?**				
Teeth-cleaning frequency				
Low frequency (≤ 0–3 times/month)	60 (38)	53 (27)	Reference	–
High frequency (≥ 2–6 times/week)	97 (62)	147 (74)	1.72 (1.09–2.69)	0.02

* Values are n (%).

† The cluster-adjusted odds ratios and *P* values are from multilevel logistic regression models with the cluster as random intercept.

**Table 4:** Self-reported problems related to the status of the teeth

	Control (*N* = 157)*	Intervention (*N* = 200)*	Odds ratio (95% CI)†	*P* value†
**How often during the past 12 months did you have toothache or feel discomfort due to your teeth?**				
Frequency of tooth ache				
Irregular	125 (81)	179 (90)	Reference	–
Frequent	29 (19)	20 (10)	0.48 (0.23–0.98)	0.04
**Have you experienced any of the following problems in the past year?**				
I have difficulty biting hard foods				
No	118 (75)	176 (88)	Reference	–
Yes	38 (24)	23 (12)	0.40 (0.21–0.77)	0.006
Don’t know	01 (0.6)	1 (0.5)	–	–
I have difficulty in chewing				
No	127 (81)	182 (91)	Reference	–
Yes	29 (18)	17 (8.5)	0.41 (0.22–0.78)	0.006
Don’t know	1 (0.6)	0	–	–
I missed school due to toothache for whole days				
No	149 (95)	188 (94)	Reference	–
Yes	8 (5.1)	10 (5.0)	0.99 (0.38–2.57)	0.98
Don’t know	0	2 (1.0)	–	–

* Values are *N* (%).

† The cluster-adjusted odds ratios and *P* values are from multilevel logistic regression models with the cluster as random intercept.

**Table 5:** Dietary habits

	Control (*N* = 157)*	Intervention (*N* = 200)*	Odds ratio (95% CI)†	*P* value†
**How frequent do you eat or drink the following? (≤ once/week = Low; > once/week = High frequency)**				
Fresh fruit				
Low frequency	95 (61)	99 (50)	Reference	–
High frequency	62 (39)	100 (50)	1.56 (0.86–2.83)	0.15
Biscuits, cakes, cream, and sweet pies				
Low frequency	133 (85)	155 (77)	Reference	–
High frequency	24 (15)	45 (23)	1.63 (0.76–3.50)	0.21
Lemonade, Coca cola, and other soft drinks				
Low frequency	144 (92)	187 (94)	Reference	–
High frequency	13 (8.3)	13 (6.5)	0.77 (0.35–1.71)	0.52
Jam and honey				
Low frequency	149 (95)	189 (95)	Reference	–
High frequency	8 (5.1)	10 (5.0)	0.98 (0.38–2.56)	0.98
Chewing gum with sugar				
Low frequency	151 (96)	190 (95)	Reference	–
High frequency	6 (3.8)	10 (5.0)	1.32 (0.47–3.73)	0.59
Sweets or candy				
Low frequency	136 (87)	176 (88)	Reference	–
High frequency	21 (13)	24 (12)	0.88 (0.42–1.81)	0.72
Milk with sugar				
Low frequency	147 (94)	174 (87)	Reference	–
High frequency	10 (6.4)	26 (13)	2.17 (0.46–10.2)	0.33
Tea with sugar				
Low frequency	134 (85)	166 (83)	Reference	–
High frequency	23 (15)	34 (17)	1.21 (0.55–2.65)	0.64
Coffee with sugar				
Low frequency	154 (98)	198 (99)	Reference	–
High frequency	3 (1.9)	1 (0.5)	0.26 (0.03–2.52)	0.24
Bushera with sugar				
Low frequency	107 (68)	129 (65)	Reference	–
High frequency	50 (32)	70 (35)	1.19 (0.44–3.25)	0.73
Sugarcane				
Low frequency	107 (68)	147 (74)	Reference	–
High frequency	50 (32)	52 (26)	0.80 (0.20–3.11)	0.75

* Values are *N* (%).

† The cluster-adjusted odds ratios and *P* values are from multilevel logistic regression models with the cluster as random intercept.

## DISCUSSION

This study demonstrates a significant effect on oral health of 5–6-year-old children after an education intervention including focus on oral health given to mothers when their children were aged 6–8 months. Less caries was first demonstrated in primary incisors when these children were 3 years old^27^ and has then persisted without any further intervention. Notably, in the current follow-up study, the intervention group experienced less caries in primary molars, less pain, and less difficulty chewing and eating compared with children in the control group.

Previous studies from rural communities in Uganda reported caries prevalence ranging from 34.5% in the age group 5–6 years^32^ to 49.2% in the age group 5–7 years^33^ and 65% in 5-years old.^34^ The prevalence of dental caries varies across rural and urban areas,^35^ and also within areas with either naturally low- or high-levels of fluoride in the drinking water. We found that more children in the intervention group (66%) reported using toothbrush to clean their teeth, compared with children in the control group (38%). The use of chewing sticks were equally frequent in both study groups. Chewing sticks, made from local trees or shrubs, offer a natural alternative to toothbrushes, with low cost and availability in LMICs,^50^ and it is frequently used in Uganda.^33^^,^^35^

The significantly higher frequency of teeth-cleaning and use of toothbrush in the intervention group is most likely the major reason for the improved oral health that we found in the intervention compared with the control group. Daily mechanical removal of tooth biofilm together with fluoride toothpaste is an important and efficient way to prevent dental caries.^36^

Our results showed that only a minority of the children had visited a dentist in the past 12 months, and many of them never had a dental visit. These findings are consistent with findings from other studies showing that the majority of the population in rural areas of Uganda do not have access to oral healthcare services, and most dental decay remains untreated.^22^^,^^33^

In many rural areas of Uganda, toothpaste is not easily accessible because many poor families cannot afford it, a fact that might explain the very low frequency of use of fluoridated toothpaste in both study groups.

The benefits of fluoride in caries prevention are generally accepted worldwide.^16^ In some parts of Africa, fluoride occurs naturally in drinking water.^37^ The water fluoride concentration in the present study area was measured when the children were 36 months, and the mean concentration was below the levels of caries preventive effect (< 0.7 ppm).^27^ On the other hand, drinking water with more than 1.5 ppm of fluoride can give rise to tooth development disturbance like dental fluorosis. The enamel defects can differ in intensity from mild to severe according to the Thylstrup and Fejerskov index.^38^ We found enamel defects in the permanent first molar consistent with mild dental fluorosis in 11 children in two villages; thus, dental fluorosis seemed not to be a big problem. Dental fluorosis is very rare in deciduous teeth and most of the studied children had primary teeth only. When fluoridated toothpaste is unavailable, fluoridation of public water supplies can be a cheap and efficient strategy to obtain optimal levels of caries prevention in the community.^10^^,^^39^^,^^40^

There is a clear association between caries and the consumption of sugar.^41^ However, we found no significant difference between the two study groups regarding dietary intakes. More than half of the children in both groups reported high intake of food and drinks containing sugar. Lifestyle changes like diet improvement is challenging, especially in poor populations with limited access to information on diet and its effect on health.

The registration of signs of removed *ebiino* (“false teeth”) was based on the photographs that by chance included the primary canine region and the results cannot be interpreted as prevalence of removed *ebiino*, but we registered possible signs of removed tooth buds in 13.7% of the children. The most common sign was missing primary canines in the lower jaw. However, at follow-up, when the children were 36 months old, a significantly reduced occurrence of removed tooth buds was reported in the intervention group compared with the control group (8.9% versus 24.7%).^27^ Few studies have reported on the prevalence of removal of primary tooth buds in rural Uganda. Nuwaha et.al found that 58.6% households in the Bushenyi district, Uganda reported that at least one child younger than 5-year-old had suffered from this practice.^42^ In 2016, Musinguzi et al. found that the prevalence of missing teeth due to removal of *ebiino* was 8.1% in rural Uganda.^43^ The practice is often performed when the child is 5–7 months old.^19^^,^^44^ At this age, the gum swelling is most evident and infants are most likely to suffer from common childhood illness (fever, diarrhea, and vomiting). Therefore, infant illnesses can easily be attributed to teething. We found no significant difference between the two study groups regarding signs of removed *ebiino*. This is probably because oral health information was not included in this study before the children were 12–16 months; consequently, many children had already been subjected to the procedure.

Many countries have introduced school-based programs, usually involving tooth brushing with fluoride-containing toothpaste, to improve oral health in children.^45^ Such programs are effective, but the prevention should start as soon as the first teeth erupt to prevent caries in the deciduous teeth. Children with caries experience in the primary dentition are more likely to develop dental caries in their permanent dentition.^46^ Since parents/caregivers have great influence on what their children eat and drink from early age, and whether they clean their teeth, educating them, such as in our RCT, seems to be a feasible and a cost-effective way to improve oral health in early childhood.^47^

The robust randomized design and the long follow-up time constitute the major strengths of this study, and is the first of its kind testing the effect of an early education intervention on oral health among children living in rural Uganda and probably in Sub-Saharan Africa. Although the total follow up-rate was acceptable (70%), the specific follow-up rates for the two study groups differed somewhat (63%-control and 76%-intervention), which may have impacted on the results. A further limitation is the indirect identification of caries using photographs and the collection of self-reported data that can be prone to bias. Our findings are, therefore, not readily comparable to other studies as the registration of dental caries was based on photographs covering primary maxillary and mandibular molars and permanent first molar if present. Since no clinical or radiographic examination was performed and only obvious cavitated lesion into the dentin seen on the photographs were registered as caries, the occurrence of caries may have been underdiagnosed. The challenge is to find an easy and cheap intervention that gives a large effect, especially in countries with limited economic and personnel resources.^48^^,^^49^ Our results shows that the maternal education intervention was effective in caries prevention.

## CONCLUSION

This study shows that dental caries and false teeth removal continue to burden children in rural areas of Uganda. Notwithstanding the limitations of this study, we conclude that maternal education intervention delivered by health personnel in early childhood managed to improve oral hygiene and give a long-term improvement of oral health. Educational interventions to parents and/or caregivers with emphasis on nutrition and oral hygiene may provide a feasible strategy to decrease progression of childhood caries and improve oral health in LMICs.

## Supplemental files


Supplemental materials
