# Supplementary material for: Long-Term Effects of a Randomized Maternal Education Trial in Rural Uganda: Implications for Child Oral Health
Source: Am J Trop Med Hyg. 2022 Sep 6;107(4):939–47. doi: 10.4269/ajtmh.22-0248 (PMC9651530; doi:10.4269/ajtmh.22-0248)
Supplement: Supplementary file 1 [file tpmd220248.SD1.pdf]

Supplementary material

Supplementary appendix S1:

The education intervention in the original cluster-randomized controlled trial

An education intervention emphasizing nutrition, hygiene (including oral hygiene) and stimulation was delivered to mothers in the intervention group. Cooking and oral hygiene demonstrations together with making of play toys to promote child stimulation were parts of the education intervention package. The intervention started when the children were between six and eight months and lasted six months in which each group of mothers received three main education sessions (with a nutrition education team) followed by monthly mother group meetings to remind them of key aspects of the intervention. Our strategy with the intervention was to promote behaviour change through providing information and prompt practice (demonstrations). The intervention is detailed below.

Nutrition education

The nutrition package was centred on PAHO/WHO guiding principles of complementary feeding of a breastfed infant (quality and quantity of complementary feeds) [1]. The main emphasis was on:

- The importance of breastfeeding and a demonstration of how to position and attach the infant to the breast.
- The need to allow emptying one breast before changing to the other breast so that the infant could benefit from both the fore and hind breast milk.
- Breastfeeding eight or more times in a day including at night.
- All mothers were asked to start complementary feeding if they had not done so, since all infants were between 6 and 8 eight months of age.
- In complementary feeding, they were advised to start with soft foods in small amounts at a time and gradually increase the portion and the thickness of the food.
- Providing food that is rich in variety of nutrients and the importance of combining a variety of foods in one dish.
- To give infants complementary foods 2-3 times a day and increase the frequency of feeding to 3-4 times a day as the child grew.
- Providing nutritious healthy snacks (such as fruit) to the infant in between the main meals.
- Interaction and responsiveness while feeding the infants by talking, smiling and

encouraging them to eat more without forcing them; to exercise patience and make feeding session a time for joy and bonding.

- To allow the infants to eat finger foods which they could hold with their hands.
- Continued breastfeeding until the child was 24 months of age.
- Breastfeeding more frequently, providing more fluids during illness (especially in diarrhoea and fever) of the infant, and giving foods that are more nutritious after recovery.

Cookery demonstrations

The cookery activities involved:

- Dishes which could combine up to 13 different foods in one obtained in their local environments.
- Inexpensive formulated recipes using locally available foods with emphasis on animal protein obtained from silverfish (*Rastrineobola argentea)* locally known as *Mukene.*
- Soy milk making, scraping meat (muscle), preparation of pumpkin seed powder and silverfish powder to incorporate in the infant's food, addition of oil/fat and sugar to porridges to increase the energy content.
- Preparation of enriched porridge recipe 1 and 2 which were enriched with the ingredients of; cooking oil, sugar, silver fish powder, milk, pumpkin seed powder and eggs; in combinations of two or more.
- Preparation of scrambled eggs preferred to the boiled eggs or omelette, which are rather hard for the infants to consume.

Hygiene education

Themes of emphasis included:

- The importance of living in a clean home environment for the good health of the family particularly the young children.
- The basic requirement to always wash hands and utensils with clean water and soap during food preparation and infant feeding.
- The prerequisite to clean food before preparation to make it free of soil and other contaminants.
- Mothers were encouraged to carry water and a piece of soap to the field/gardens to wash hands before feeding the infants.

- Mothers were warned on giving leftover foods to the infants, since safety of such food was not possible and safe for the infants to consume later.
- Licking spoons as they fed the babies (to test the temperature) was discouraged to avoid transmission of infections from the mother to the infant.

Oral health education

- Oral hygiene was emphasized, which included cleaning the mouth of the baby with a clean cloth and warm water. Children and the entire family were later given tooth brushes with emphasis on how to use, clean and store them.

- In oral hygiene, emphasis was on: (a) the importance of cleaning the oral cavity and specifically teeth; (b) the distribution of toothbrushes to the children and all members of the family; (c) demonstration of the cleaning of the oral cavity of the infant with clean, boiled water and a soft tooth brush which we had supplied. In addition, the mothers were encouraged to: (d) brush the infant's teeth with clean water at least twice a day, especially before going to bed; (e) clean the brushes after use before storing them safely in a clean container, preferably with a cover, and (f) not to share the tooth brushes.

The booster sessions were shorter and usually lasted 3- 4 hours. Almost all the mothers (94%) in each group would attend the sessions with their babies. *Child stimulation*

The child play and stimulation emphasized:

- The importance of age graded child play activities and the role of mothers, other family members to engage in child stimulation.
- The significance of play to promote healthy development of the child.
- Explanation of the three development domains (cognitive, language and motor domains).

We explained to the mothers that the aim of play was to develop imagination creativity and social skills in the child [2]. The mothers were encouraged to use "name and identify" child's body parts to facilitate the child's understanding during his/her daily routine related to his body [3]. Practically, mothers engaged children in some of child play activities such as hiding favourite items for children to find; screwing and unscrewing bottles and imaginary play. Mothers also hand-made "easy to make" toys (from local materials) which were recommended as appropriate for children; shakers, empty transparent bottles with screws and food pellets inside, baby dolls made from cloth or banana fibres.

Language development was defined as verbal and non-verbal communication (expressive and receptive language) [4]. “We Talk” slogan was used to show mothers the importance of talking to the child so that they learn to talk back and in the process develop language skills [4]. Mothers were encouraged using communication development aides such as imitation, roleplaying games, songs and music, to facilitate the child’s ability to communicate emotions, thoughts, needs and interests [5,6]. The mothers were encouraged to set aside time to purposefully talk to the children, call them by their name and to respond to them in word and/by gesturing; mention household and personal items while pointing at them, naming domestic animals, imitating their words and actions.

For motor development, the “Learn whereas playing” slogan was emphasized. The concept of gross motor skills was explained as the use of coordination and control of the body to facilitate the development of security, speed, and accuracy [7] in daily performance of tasks in a child’s life (larger movements like walking and kicking). Fine motor skills were defined as the ability to perform complex skills for more proficient tasks of daily living [5] (smaller movements like writing, tying shoelaces, and unbuttoning clothes). The following activities were emphasized:

- Giving child items to hold with their fingers, for example handing a pencil and paper for them to scribble.
- Matching lids with same size colour and shape games.
- Threading with beads
- Poking straws into holes.
- Stacking cups

The recommended toys included balls, bottle lids, cups, big beads, threads, ropes, shakers, pencils and paper. Furthermore, the mothers were encouraged to empower each other, by meeting regularly in their groups to practice and evaluate their childcare skills. We also advised them to be active with their sub-county activities for easy identification by government programs targeting women.

Booster sessions of the educational components after the intervention period

To prolong the effects of, and adherence to, the education intervention after the 6-months’ intervention period had ended and until the children were aged 36 months, we administered booster sessions to groups of 6-12 women from the original trial cohort of 511 women. These sessions (each lasting about 6 hours) were provided by the education team every third month and started three months after end of the intervention period, hence a maximum of 8 booster sessions were given. The sessions were reminders of the education activities taught during the

intervention period and re-emphazised the importance of (i) making nutritious meals; (ii) hand-washing and hygienic preparations, and (iii) child stimulation. During the follow-up period to child age of 36 months, the field-workers visited the mothers on three occasions to encourage them to continue these oral hygiene practices. Lost or damaged tooth brushes were replaced also during these visits. After the child age of 36 months, the mother/child pairs had no contact with the study team until the current follow-up study was conducted when the children had reached the age of 60-72 months.

Routine health care practices

The intervention group received routine health care and the education intervention while the control group received only routine health care. The routine health care consisted of the recommended regular anthropometric measurements, immunizations, deworming, vitamin A supplementation, malaria-prophylaxis and iron-deficiency anemia prevention. Importantly, when the children were aged 20-24 months we found that mothers in the intervention group had gained significantly more knowledge and better practices related to child feeding, hygiene and stimulation [8] compared to the control mothers, indicating that the contents of our education intervention differed markedly from routine health care.

Sample size calculation

In the original cluster-randomized controlled trial, the sample size calculation was based on the primary outcome which was height-for-age z-score (HAZ) at child age of 20–24 months. The mean ± SD for HAZ is 0.0 ± 1.0 in a healthy population. We defined a difference of 0.3 SD in HAZ between the intervention and control group as clinically relevant, corresponding to about half a percentile in HAZ [9]. To detect a change of 0.3 SD in HAZ with a significance level of 5% and a power of 80%, 176 children were required per group. Fifty-one children per sub-county were included presuming 10 sub-counties as clusters and an intra-cluster correlation of 0.01 [10]. To also account for dropouts etc., we ended up by including 511 mother–children pairs, and the assessment was by intention-to-treat. Authors GM and PA generated the random allocation sequence, enrolled the study participants, and assigned participants to interventions.

References

1. PAHO/WHO, *Guiding Priciples for Complementary Feeding of the Breastfed Child.* Division of Health Promotion and Protection, 2003(Washington D.C.).
2. Bruner, J.S. and H. Haste, *Making Sense (Routledge Revivals): The Child's Construction of the World*. 2010: Routledge.

3. Chang, S.M., et al., *Integrating a parenting intervention with routine primary health care: a cluster randomized trial.* Pediatrics, 2015. 136: 272-280.
4. Vos, R.C., et al., *Developmental trajectories of receptive and expressive communication in children and young adults with cerebral palsy.* Dev Med Child Neurol, 2014. 56: 951-959.
5. Hartinger, S.M., et al., *Impact of a child stimulation intervention on early child development in rural Peru: a cluster randomised trial using a reciprocal control design.* J Epidemiol Comm Health, 2017. 71: 217-224.
6. Wolf, J., et al., *Impact of a child stimulation intervention on early child development in rural Peru: a cluster randomised trial using a reciprocal control design.* 2017. J Epidemiol Community Health, 71: 217-224.
7. Donnelly, J.E., et al., *Physical activity, fitness, cognitive function, and academic achievement in children: a systematic review.* Med Sci Sports Exerc, 2016. 48: 1197.
8. Muhoozi, G.K., et al., *Nutrition, hygiene, and stimulation education to improve growth, cognitive, language, and motor development among infants in Uganda: A cluster-randomized trial.* Matern Child Nutr, 2018. 14: e12527.
9. Ong, K. K., et al., *Association between postnatal catch-up growth and obesity in childhood: Prospective cohort study.* BMJ, 2000. 320: 967–971.
10. Campbell, M. J., et al., *Developments in cluster randomized trials and Statistics in Medicine.* 2007. Statistics in Medicine, 26: 2–19.

Supplementary appendix S2:

The oral health questionnaire

We used the WHO – oral health questionnaire, children's version [1]. Self-reported oral health was assessed by asking several questions:

1. *How would you describe the health of your teeth, and how would you describe the health of your gums?* The response alternatives were: "excellent", "very good", "good", "average", "poor", "very poor" and "I don't know." During analysis, the categories were dichotomized into "good" (excellent, very good, good) and "poor" (average, poor, very poor). "I don`t know"-responses were recoded as missing.
2. *How often during the past 12 months did you have toothache or feel discomfort due to your teeth?* The response categories were: "often", "occasionally", "rarely", "never" and "I don't know#. The categories were dichotomized into "frequent" (often, occasionally) and "irregular" (rarely, never). "I don't know"-responses were recoded as missing.
3. *How often did you go to the dentist during the past 12 months?* The response alternatives were: "once", "twice", "three times", "four times", "more than four", "I had no visit to dentist during the past 12 months", "I have never received dental care/visited a dentist" and "I don't know/don't remember". The response categories were dichotomized into "one or more visits" and "no visit". "I don't know/don't remember"-responses were recoded as missing. If the child had visited a dentist during the last year, the response options were: "pain or trouble with teeth", "gums or mouth", "treatment/follow-up treatment", "routine check-up of teeth/treatment" and "I don't know/don't remember".
4. *How often do you clean your teeth?* The response options were: "never", "several times a month (2-3 times)", "once a week", "several times a week (2-6 times)", "once a day" and "two or more times a day". The categories were dichotomized into "low frequency" (never, several times a month, once a week) and "high frequency" (several times a week, once a day, two or more times a day).
5. *Do you use any of the following to clean your teeth or gums; toothbrush, wooden toothpicks, plastic toothpicks, thread (dental floss), charcoal, chewstick/miswak or other?* The response categories were: "yes" or "no" for each item.

6. The participants were then asked about their use of toothpaste with two questions: (i) *Do you use toothpaste to clean your teeth* and (ii) *do you use toothpaste that contains fluoride?* The answer categories were: "yes" or "no" for both questions and "I don't know" was added for the last question.
7. The children and their parents were also asked: *Because of your state of your teeth and month, have you experienced any of the following problems during the past year: (a) Toothache or discomfort caused by my teeth forced me to miss classes at school or miss school for whole days, (b) I have difficulty biting hard foods, and (c) I have difficulty in chewing.* The responses were: "yes", "no" and "I don't know" to each item.
8. *How often do you eat or drink any of the following foods, even in small quantities: Fresh fruit, biscuits, cakes, cream cakes, sweet pies, buns, lemonade, Coca Cola, other soft drinks, jam/honey, chewing gum containing sugar, sweets/candy, milk/tea/coffee with sugar, bushera with sugar and sugarcane?* The response categories were: "never", "several times a month", "once a week", "several times a week", "every day" and "several times a day". The categories were dichotomized into "low frequency" (never, several times a month, once a week) and "high frequency" (several times a week, every day, several times a day).

Reference

World Health Organization, Oral Health Questionnaire for Children. Available at: https://www.who.int/oral_health/publications/pepannex8sohqchildren.pdf. Accessed 2022.
